# Global geochemical fingerprinting of plume intensity suggests coupling with the supercontinent cycle

**DOI:** 10.1038/s41467-019-13300-4

**Published:** 2019-11-21

**Authors:** Hamed Gamal EL Dien, Luc S. Doucet, Zheng-Xiang Li, Grant Cox, Ross Mitchell

**Affiliations:** 1 Earth Dynamics Research Group, The Institute for Geoscience Research (TIGeR), School of Earth and Planetary Sciences, Curtin University, GPO Box U1987, Perth, WA 6845 Australia; 2 Geology Department, Faculty of Science, Tanta University, 31527 Tanta, Egypt

**Keywords:** Geochemistry, Geodynamics, Petrology

## Abstract

Plate tectonics and mantle plumes are two of the most fundamental solid-Earth processes that have operated through much of Earth history. For the past 300 million years, mantle plumes are known to derive mostly from two large low shear velocity provinces (LLSVPs) above the core-mantle boundary, referred to as the African and Pacific superplumes, but their possible connection with plate tectonics is debated. Here, we demonstrate that transition elements (Ni, Cr, and Fe/Mn) in basaltic rocks can be used to trace plume-related magmatism through Earth history. Our analysis indicates the presence of a direct relationship between the intensity of plume magmatism and the supercontinent cycle, suggesting a possible dynamic coupling between supercontinent and superplume events. In addition, our analysis shows a consistent sudden drop in MgO, Ni and Cr at ~3.2–3.0 billion years ago, possibly indicating an abrupt change in mantle temperature at the start of global plate tectonics.

## Abstract (web summary)

The links between plate tectonics and deep mantle structure remain unclear. Here, the authors demonstrate that transition elements (Ni, Cr, and Fe/Mn) in basaltic rocks can be used as a tool to trace plume-related magmatism through Earth history, and their results indicate the presence of a direct relationship between the intensity of plume magmatism and the supercontinent cycle.

## Introduction

The plate tectonic theory developed last century works well on Earth’s outer shell, but how this system interacts with mantle plumes, and if they are part of the same geodynamic system in Earth history, remains unclear. Seismic studies have revealed that Earth’s present-day lower mantle is dominated by two antipodal large low shear velocity provinces (LLSVPs), also known as the African and Pacific superplumes, surrounded by high-velocity zones with subducted cold slabs^1^. It has been further shown that almost all known mantle plumes since ~300 million years ago (Ma) were generated atop or near the edges of these two LLSVPs^2,3^. On the other hand, the assembly and breakup of supercontinents are controlled by global-scale mantle dynamics and constant feedback between surface and deep mantle processes^4^. It has been further established that the African LLSVP (whether or not in its present geometry) was located underneath the supercontinent Pangaea ca. 300 Ma^5^, and that there was a close link between mantle plumes and Pangaea breakup^6^. However, how long the LLSVPs have been present, how such LLSVPs interact with tectonic plates in Earth history, and whether they are fixed in the deep mantle^1,3,7^ or part of a dynamic system associated with the supercontinent cycle since at least the Proterozoic^8–11^, remain topics of debate. Tracing mantle plume signatures throughout Earth history is fundamental for answering those questions and testing the stable vs. dynamic/cyclic nature of the LLSVPs, and thus achieving a better understanding of the coupling between Earth’s mantle dynamics and plate tectonics.

Basaltic magmatism can be used to probe mantle evolution throughout Earth history^12,13^. Such mafic magmatism is mostly generated in three main tectonic settings: mantle plume, mid-ocean ridge, and subduction zone (arc). Geochemical and isotopic characteristics of basalts generated in such settings can identify, or “fingerprint”, the processes and sources of magma generation from different parts of the mantle^12^. Where mid-ocean ridge basalts (MORBs) and arc-related basalts (ARBs) represent melts generated within the sub-oceanic and sub-arc upper mantle, respectively^14–17^, plume basalts, such as oceanic island basalts (OIBs), oceanic flood basalts, and continental large igneous provinces (LIPs) commonly involve deeper mantle processes in which LLSVPs may provide both additional heat and some melt materials^12,18,19^.

Incompatible trace elements, particularly high-field strength elements (HFSEs) and their ratios, are widely used to monitor and discriminate between mantle domains and tectonic environments for basaltic magmatism, and to trace plume signatures^15,16,20^. However, as HFSEs can be affected by various processes, such as source contamination, magma mixing, crust-magma interaction, and high-grade metamorphism, the validity of using such an approach to discriminate tectonic settings has been questioned^21,22^. One reason for this is that the low partition coefficients (<1) of HFSEs with Fe–Mg silicate minerals and spinel^12,23^—the main components of basaltic magmatism—make HFSEs easily redistributed by the aforementioned processes. On the other hand, transition elements have partition coefficients with Fe–Mg silicate minerals and spinel >1, making them highly insensitive to post-formation processes^23–25^. The behavior of the first-row transition elements, especially Ni and Cr (highly compatible) and their ratios to less compatible ones (Co and Zn), is strongly melt-composition dependent^12,23^ and highly sensitive to the earliest magmatic differentiation stages^26^. Thus, the abundance of Ni and Cr may track the nature of basaltic magmatism generated from different parts of the mantle.

Here, we demonstrate that Ni and Cr contents constitute an excellent tool for discriminating between plume- and non-plume-related (MORBs and ARBs) magmatism. In addition, by using the statistical bootstrapping method on the global geochemical database of basaltic rocks, we show that the transition elements (Ni, Cr, and Fe/Mn, i.e., plume intensity) exhibit systematic short-term variations that coincide with the supercontinent cycle, which could potentially suggest a dynamic coupling between first-order mantle structure (e.g., LLSVPs) and plate tectonics.

## Results

### Ni and Cr as tracers for mantle plume products

Magnesium content and its ratio to other elements (i.e., Mg/Fe) are commonly used as tracers for the differentiation of silicate rocks and an indicator for mantle temperature^14,15,27^. Geochemical modeling using MgO content revealed the ambient mantle temperature below the ridges and sub-arc to be ~1350 °C, but typically thermal anomalies of ~200–300 °C over the ambient mantle temperature are expected for mantle plumes^14,15,27–29^. As transition elements (i.e., Ni and Cr) behave similarly to Mg during mantle melting, Ni and Cr should also be highly sensitive to mantle temperature, and can thus be used for distinguishing between plume and non-plume mantle melt products. To illustrate this point, we compare the global databases (see the Methods section and Supplementary Data 1) of mantle melts with a range of MgO content and potential mantle temperature of melting^14^, such as komatiites (>18 wt% MgO and T ~1600 °C), picrites (>12 wt % MgO and T ~1500 °C; from OIBs and LIPs), and normal basalts (<12 wt % MgO and T ~1350 °C; from present-day MORBs and ARBs)^14,15,30,31^ to their corresponding Ni, Cr, Ni/Co, and Cr/Zn contents and ratios, respectively (Supplementary Fig. 1). Strikingly, those transition elements have a positive correlation with MgO content and can, therefore, be used for discriminating komatiites and picrites (representing plume magmatism) from normal basalts (representing non-plume magmatism). Plume magmatism is characterized by Ni > 200 ppm, Cr > 500 ppm, and Ni/Co and Cr/Zn both >8 (Supplementary Fig. 1).

**Fig. 1:**
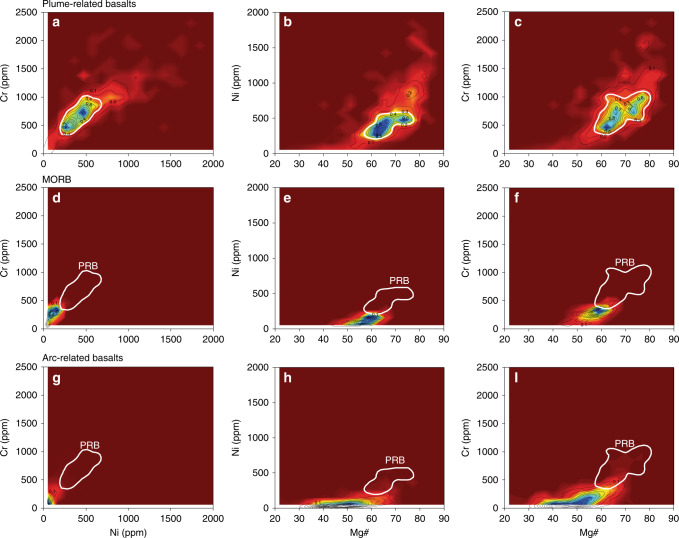
Density plot of Ni, Cr, and Mg# for different basaltic magmatism types. Panels **a**, **b**, and **c** are density plots of plume-related basalts (PRB) for Ni (ppm) vs. Cr (ppm) and Mg# (100 × MgO/ (MgO + FeO_T_) vs. Ni (ppm) and Cr (ppm), respectively. Panels **d**–**f** are density plots for mid-ocean ridge basalts (MORB) and **g**–**i** panels are for arc-related basalts. Data sources: Georoc and EarthChem (see Methods and Supplementary Data 1). Black contours define the data density values from high density (blue) to low density (red). The white contour represents 70% of the PRB data

**Fig. 2:**
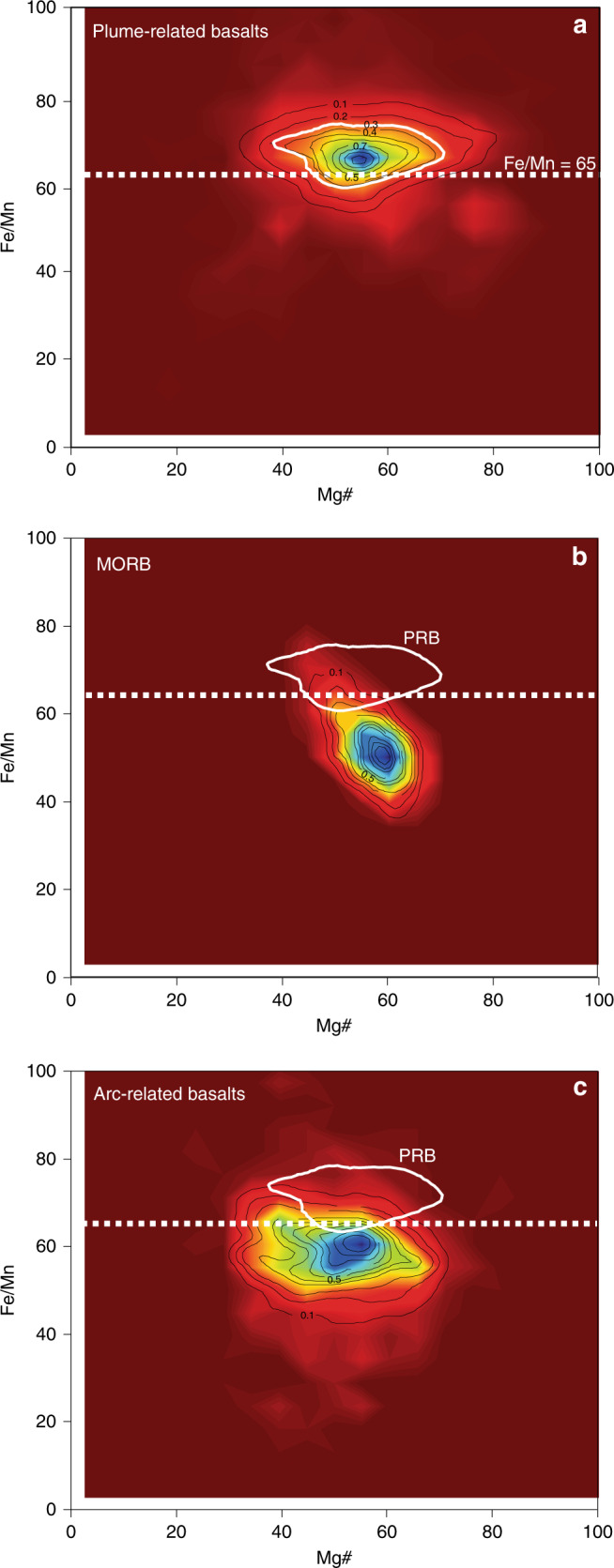
Density plot of Fe/Mn vs. Mg# for different basaltic types. **a** Plume-related basalts (PRB); **b** mid-ocean ridge basalts (MORB); **c** arc-related basalts. Data sources: Georoc and EarthChem (see Methods). Black contours define the data density values from high density (blue) to low density (red). The white contour represents 70% of the PRB data

To further verify the inferred plume characteristics, we produced covariation plots for Ni and Cr, and Mg# (100 × MgO/(MgO + FeO_T_) vs. Ni and Cr of basalt datasets (45 wt% <SiO_2_ <53 wt% and Na_2_O + K_2_O < 5 wt%)^32^ from Cenozoic OIBs and LIPs and present-day MORBs and ARBs (Fig. 1; Supplementary Fig. 2 and Supplementary Data 1). Much like komatiites and picrites, 70% of the plume basalt (PB) datasets have Ni >150 ppm and Cr > 300 ppm, whereas non-plume magmatism (MORBs and ARBs) are mostly below these limits. Thus, we argue that high Ni and Cr contents in basalts implies a plume signature. In addition, consistent with previous studies of some OIBs from the Pacific superplume^33,34^, we found that plume basalts (OIBs and LIPs) generally have Fe/Mn > 65, clearly higher than that of MORBs and ARBs (Fig. 2).

**Fig. 3:**
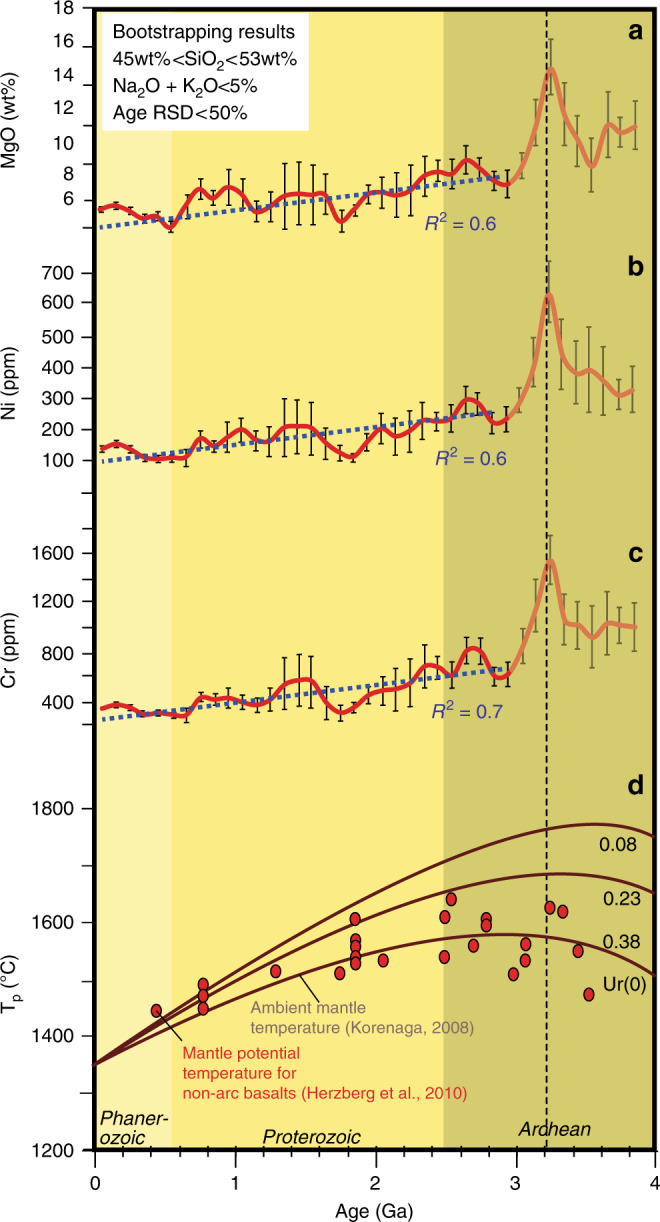
Time evolution of global mean MgO, Ni, and Cr contents after a bootstrap resampling of the selected basaltic database. The blue dashed lines in **a**–**c** are regression lines for data between 0 to 3.0 Ga that reveal first-order secular decreases. Error bars in **a**–**c** show 2 s.e.m. (standard error of the mean) uncertainties. **d** Secular cooling of Earth’s mantle based on non-arc basalts^28^ and thermal modeling^43^. Urey ratios (Ur) are shown: 0.08 (upper curve), 0.23 (middle curve), and 0.38 (lower curve)

**Fig. 4:**
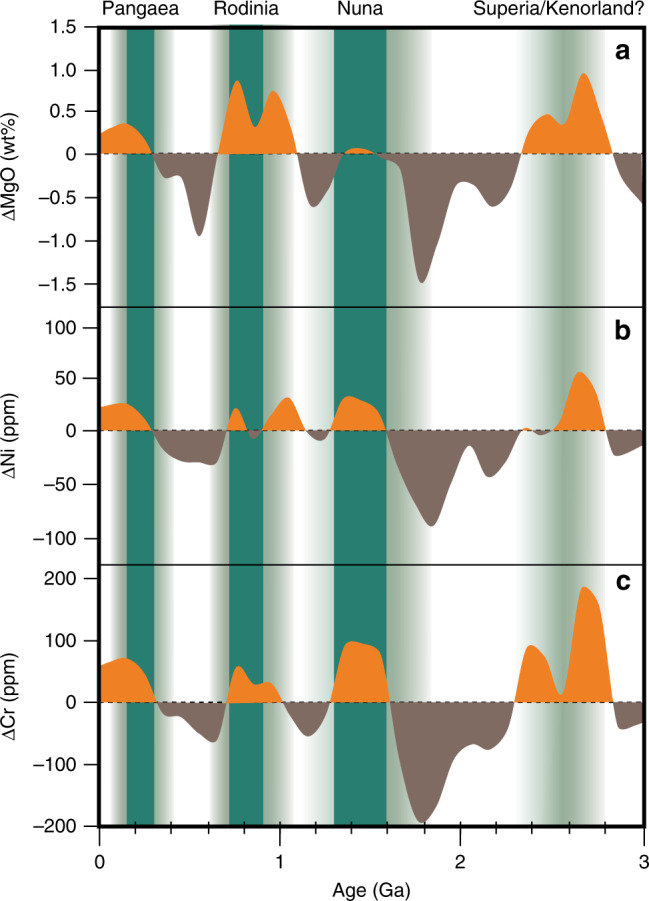
Variability of global mean MgO, Ni, and Cr in basalts after detrending the linear secular decreases. The linear secular decreases used are those shown with blue dash lines in Fig. 3. The plot shows major positive peaks at ~2.8–2.3 Ga, ~1.6–1.3 Ga, ~1.0–0.7 Ga, and ~0.3–0.0 Ga, broadly consistent with the tenures and break-up times of post-2 Ga supercontinents. The durations of supercontinent tenure^53,54^ are marked with solid green vertical bars, e.g., at ca. 320–170 Ma (Pangea), 900–700 Ma (Rodinia), 1600–1300 Ma (Nuna), but the occurrence of Kenorland during 2700–2300 Ma is highly uncertain (thus shown in faded green). The assembly and break-up of supercontinents are generally prolonged and multistage processes, which are marked by gradual green shading

Based on these observations, we use MgO, Cr, and Ni contents to filter data from ∼41,000 samples (Supplementary Figs. 3, 4 and Supplementary Data 2) in the global databases (Georoc and EarthChem repositories) in order to trace plume signature throughout Earth history (see the Methods section). The bootstrapped data (see Methods) reveal a first-order decrease in MgO, Cr, and Ni content in basalts since the Archean eon^29,35^ (Fig. 3), plus second-order variations within the Proterozoic and the Phanerozoic (Fig. 4). To further interrogate the second-order variations, we detrended the linear secular decreases in each dataset (Fig. 4a–c; Supplementary Fig. 4 and Supplementary Table 1).

## Discussion

### Time variations in mantle plume intensity

The highly compatible transition elements Ni and Cr and their ratios to less compatible ones (e.g., Ni/Co and Cr/Zn), plus Fe/Mn and the previously widely used MgO, are good discriminants for mantle plume magmatism because they all indicate higher mantle melt temperature. As basaltic magmatism represents the direct melt products of Earth’s mantle, its composition potentially records Earth’s mantle evolution since 4 billion years ago (Ga). Our analysis revealed a first-order decrease in MgO, Ni and Cr since the Archean similar to that shown by Keller and Schoene^29,35^ (Fig. 3a–c), readily explainable by Earth’s secular cooling (including the lowering of the mantle potential temperature)^15,28,29,35^.

The second-order variability of MgO, Ni, and Cr, previously largely ignored^29,35,36^, is more intriguing. The curves consistently show major positive peaks at ~2.8–2.3 Ga, ~1.6–1.3 Ga, ~1.0–0.7 Ga, and ~0.3–0.0 Ga. The most straightforward interpretation of these second-order variations is that they reflect changes in mantle potential temperature/degree of melting (see Fig. 4 of Ref. ^29^), which may be a consequence of either mantle plumes, or thermal insulation in the upper mantle^37^, or both. Plumes are possibly a more dominant factor because they allow the higher Ni (>150 ppm) and Cr (>300 ppm) contents of the basalts to be sourced from the fertile lower mantle peridotites (i.e., Ni = 2500–3200 ppm and Cr = 2600–7500 ppm)^14,38,39^ (Fig. 1a–c) rather than the depleted upper mantle peridotites (i.e., Ni = 1960 ppm and Cr = 2500 ppm)^40^ (Fig. 1d–f). This interpretation is supported by their anomalously high Fe/Mn of >65 (Fig. 2), a well-accepted characteristic of plume basalts^33,34^, as well as high abundances of Ni and Cr (Fig. 1).

### Implications for mantle dynamics coupled with the supercontinent cycle

Our results show a general temporal consistency between the MgO, Ni, and Cr peaks/anomalies (i.e., plume intensity/activities) and the occurrence of known supercontinents, suggesting a clear positive correlation between mantle plume intensity and supercontinent tenure (Fig. 4). Such results allow us to speculate on the fixed vs. dynamic models for the two LLSVPs in the lower mantle. In the fixed LLSVPs model^1,3,7^, the LLSVPs are stable features anchored to the core-mantle boundary (CMB) since early Earth. Their positions and shapes would therefore not have been linked to the subduction girdle and thus to plate motion in general^8–11^. As such, one would expect the formation of plume basalts to be stochastic in Earth history, implying a semi-uniform occurrence of plume magmatism over geological time, i.e., plume intensity unrelated to the supercontinent cycle. Alternatively, if supercontinents indeed preferentially form on the global subduction girdle (cold downwelling mantle) as suggested by some^41^, and both the antipodal LLSVPs and the subduction girdle are fixed and long-lived features, then one would expect an anticorrelation between plume intensity and supercontinental tenure (we note that the pre-Cretaceous global plume record is dominated by continental LIPs). In contrast, according to the dynamic LLSVPs model^8,9^, the formation of antipodal LLSVPs is linked to circum-supercontinent subduction that leads to the formation of the subduction girdle; the subduction girdle subsequently divides the hot and dense lower mantle into the two antipodal LLSVPs^8,9^. Such a dynamic LLSVP model therefore predicts an increase in the intensity of plume magmatism during the tenure and breakup stage of the supercontinent, and thus a periodicity positively correlated with the supercontinent cycle.

The global plume intensity record we reported here (Fig. 4) is inconsistent with either predictions of the fixed LLSVPs model, i.e., it shows neither a semi-uniform time distribution, nor an anticorrelation with the supercontinent tenure. The observed positive correlation between plume intensity and supercontinent cycle cannot be viewed as mere coincidence. In the absence of viable alternative explanations, we suggest the coupled supercontinent-mantle plume records as evidence supporting the dynamic LLSVP model. Indeed, it would be challenging to keep the LLSVPs fixed to the CMB because a constantly evolving subduction geometry changes the deposition of the subducted slabs above the CMB^42^.

Furthermore, our analysis shows a consistent sudden drop in MgO, Ni, and Cr at ~3.2–3.0 Ga (Fig. 3a–c), interpreted to indicate an abrupt change in mantle temperature. These results are comparable with the petrological estimation of mantle potential temperature using non-arc basalts^28^, as well as Earth’s secular cooling predicted by parameterized convection models^43^ (Fig. 3d). We speculate that this interpreted dramatic drop in mantle temperature may indicate the initiation of global plate tectonics, where the subduction-driven whole-mantle convection enhanced the heat flux out of the core and mantle. The sharp peaks at ca. 3.2 Ga (Fig. 3a–c), also shown in the mantle potential temperature estimations based on non-arc basalts (red dots in Fig. 3d)^28^, possibly reflect a dramatic build-up in mantle temperature before the initiation of plate tectonics, when there was a lack of a mechanism for efficient heat flux out of the mantle. A ~3.2–3.0 Ga emergence of plate tectonics would be broadly consistent with the sedimentary, igneous, metamorphic, and palaeomagnetic records^26,36,44^.

To summarize, geochemical tracers for plume basalts (Ni, Cr, and Fe/Mn) indicate the presence of a coupling between global plume intensity and supercontinent cycle. Such results are consistent with the suggested dynamic LLSVP model^8–10^ that links the formation, position, and evolution of LLSVPs with that of supercontinents. Recent numerical modeling^45–48^, paleomagnetic^49,50^, and seismic^51,52^ work are also in general agreements with the geodynamic LLSVP model.

## Methods

### Database compilation

We compiled a global database of komatiites (~3000 samples) and picrites (~1650 samples), covering major- and trace elements (particular transition elements such as Ni, Cr, Co, Zn, Cu, and Sc) from the Georoc (http://georoc.mpch-mainz.gwdg.de/georoc/) and EarthChem (https://www.earthchem.org/) repositories (Supplementary Data 1). The komatiite and picrite samples in the database have ages ranging from Archean to present-day. We made a manual double-check of data quality against the original references to choose only komatiite (>18 wt% MgO, SiO_2_ < 52 wt%, and total alkali (K_2_O + Na_2_O) < 2 wt%) and picrite (>12 wt% MgO, SiO_2_ < 52 wt% and total alkali (K_2_O + Na_2_O) < 3 wt%) compositions^14,15,30,31^ for the plots of Supplementary Fig. 1. The present-day mid-ocean ridge basalts (MORBs) and arc-related basalts (ARBs), used in Figs. 1, 2 and Supplementary Figs. 1, 2, are assembled from the same repositories and filtered for samples with basaltic composition (i.e., 45–53 wt% SiO_2_, MgO < 12 wt%, and total alkali (K_2_O + Na_2_O) < 5 wt%^14,15,31,32^) and comprehensive major- and transition elements datasets including Ni, Cr, Co, Zn, Cu, and Sc. The present-day MORBs whole rock and glass database (~1200 samples) includes Atlantic, Pacific, and Indian mid-ocean ridges; the ARBs database (~5300 samples) includes oceanic-arc basalts, such as the Izu–Bonin–Mariana, Tonga, Sunda, Aleutian, Kermadec, New Hebrides, Kurile, and Lesser Antilles arcs, and continental-arc basalts such as that of the Andean, Cascades, and Central American arcs (Supplementary Data 1). The oceanic island basalts, oceanic flood basalts, and large igneous provinces database (~6200 samples) includes mainly Cenozoic basalts, such as the Hawaiian islands, Canary islands, Cape Verde islands, Society islands, St. Helena Chain, Deccan, Afro-Arabia, North Atlantic, Columbia River, Caribbean-Colombian, Hess rise, and Manihiki Plateau basalts (Supplementary Data 1).

### Data bootstrap resampling

A geochemical database of major and transition elements of basalts (~41,000 samples), with ages ranging from 3.8 Ga to present-day, was extracted from the Georoc and EarthChem repositories (Figs. 3, 4; Supplementary Data 2), including age estimates and geospatial sample locations for each sample. Data with unknown age or sample location, suspicious data with major elements totaling >100%, and data from ultramafic cumulates or komatiites, have been manually filtered out. The database mainly consists of samples with a basaltic composition (i.e., 45–53 wt% SiO_2_ and total alkali (K_2_O + Na_2_O) < 5 wt%^31^) and age uncertainties below 50% relative standard deviation (RSD). To obtain an optimal estimate of composition distribution of Ni, Cr, and MgO in mantle-derived melts and minimize sampling and preservation bias, we did a weighted bootstrap resampling of the database following the method of Keller and Schoene^35^ using the Matlab MIT open-source code available at https://github.com/brenhinkeller/StatisticalGeochemistry.

## Supplementary information

Peer Review File

Supplementary Information

Description of Additional Supplementary Files

Supplamentery Data 1

Supplamentery Data 2

## Data Availability

All the data that are necessary for evaluating the findings of this study are available within this article and it’s Supplementary Information.
