# Author Correction: Global geochemical fingerprinting of plume intensity suggests coupling with the supercontinent cycle

**DOI:** 10.1038/s41467-020-16730-7

**Published:** 2020-06-05

**Authors:** Hamed Gamal EL Dien, Luc S. Doucet, Zheng-Xiang Li, Grant Cox, Ross Mitchell

**Affiliations:** 1 Earth Dynamics Research Group, The Institute for Geoscience Research (TIGeR), School of Earth and Planetary Sciences, Curtin University, GPO Box U1987, Perth, WA 6845 Australia; 2 Geology Department, Faculty of Science, Tanta University, 31527 Tanta, Egypt

**Keywords:** Geochemistry, Geodynamics, Petrology

Correction to: *Nature Communications* 10.1038/s41467-019-13300-4, published online 21 November 2019.

The original version of this Article omitted from the author list the 4th and 5th authors, Grant Cox and Ross Mitchell, who were both at Curtin University. Consequently, the corrected version of the Acknowledgements removes the following from the original version: ‘and Ross Mitchell’. In addition, the following was added to the Author Contributions: ‘G.M.C. and R.M. were involved in an early attempt of this study.’. This has been corrected in both the PDF and HTML versions of the Article.

