# Supplementary material for: Global geochemical fingerprinting of plume intensity suggests coupling with the supercontinent cycle
Source: Nat Commun. 2019 Nov 21;10:5270. doi: 10.1038/s41467-019-13300-4 (PMC6872659; doi:10.1038/s41467-019-13300-4)
Supplement: Supplementary file 2 — Supplementary Information [file 41467_2019_13300_MOESM2_ESM.pdf]

## Supplementary information for:

# Global geochemical fingerprinting of plume intensity suggests coupling with the supercontinent cycle

**By Gamal EL Dien et al.**

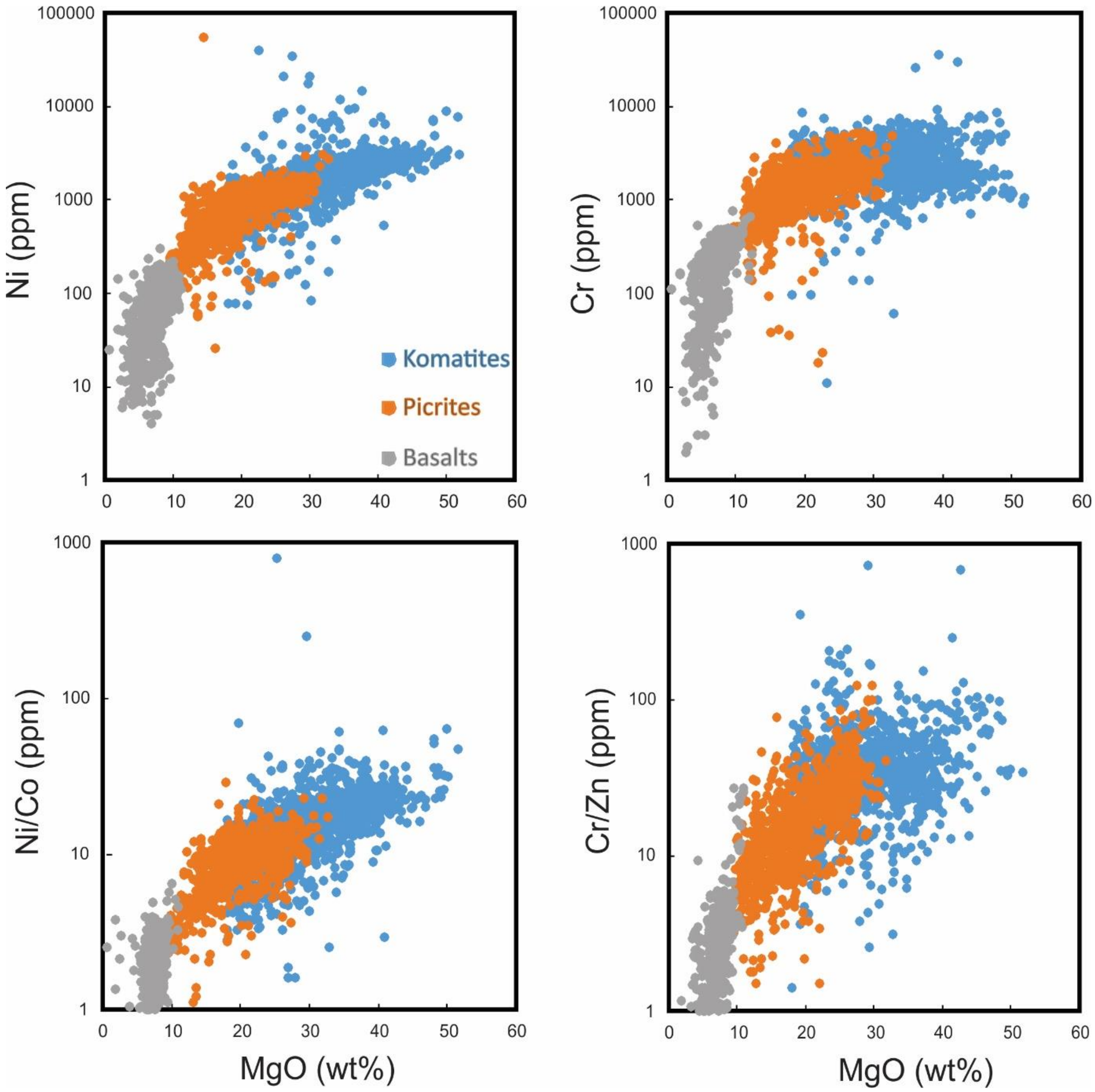


**Supplementary Figure 1**: Covariation of MgO vs Ni, Cr, Ni/Co and Cr/Zn for different mantle magmatic products (komatiites, picrites and basalts). Data source: Georoc and EarthChem.

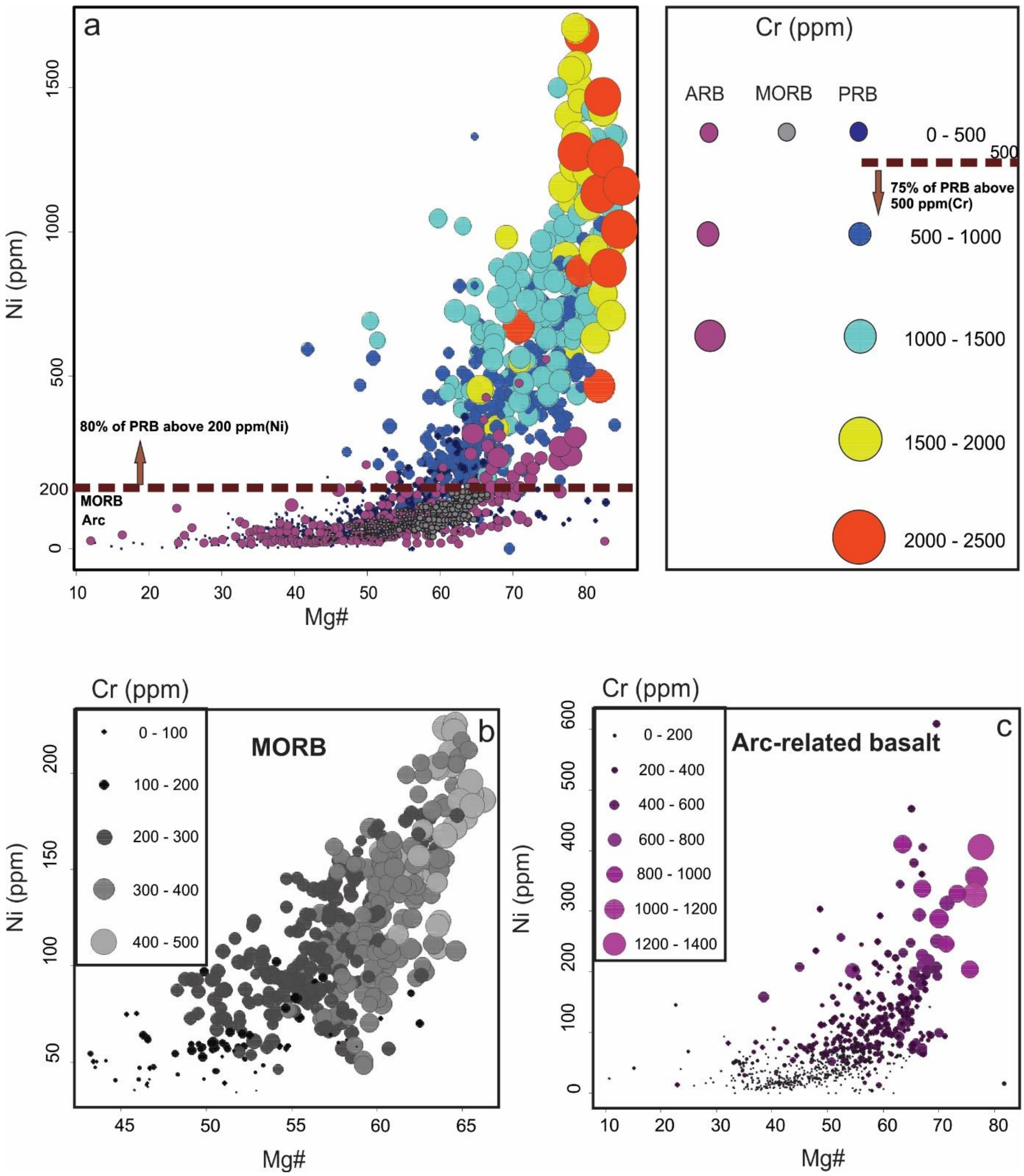


**Supplementary Figure 2**: Covariation plots of Mg# (100*MgO/ (MgO+ FeO) vs. Ni with the size of the symbol proportional to Cr content in each samples for plume-related basalts (PRB) (a), mid-ocean ridge basalts (MORB) (b) and arc-related basalts (c). Data sources: Georoc and EarthChem.

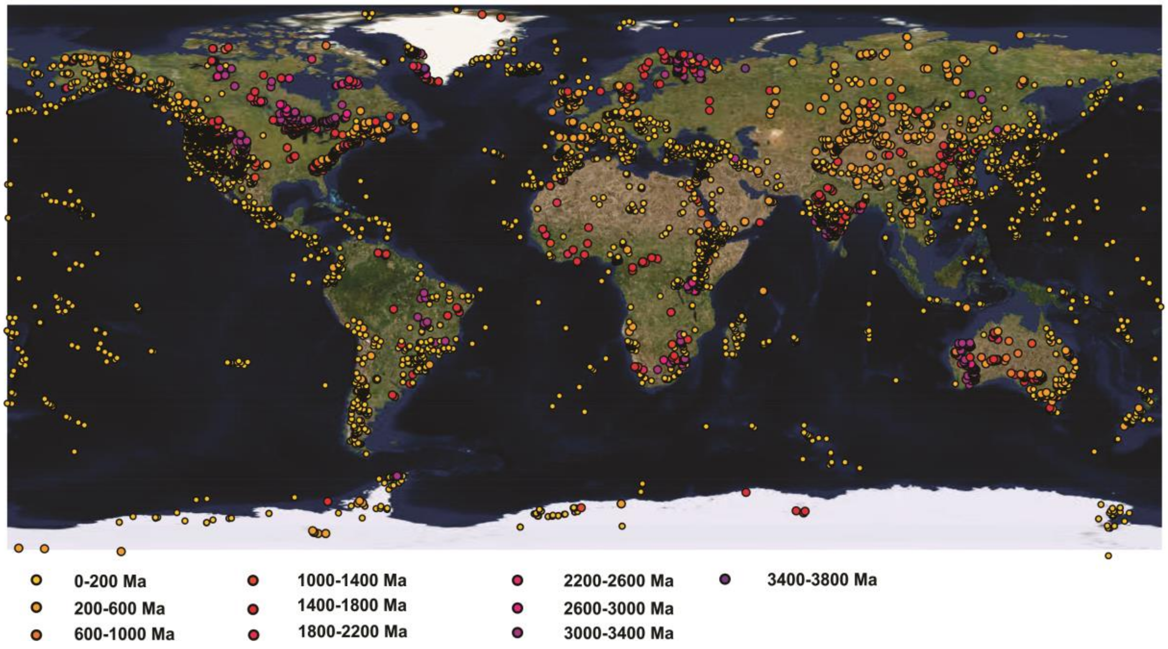


**Supplementary Figure 3**: Geographic distribution map of basaltic datasets used for bootstrap resembling and the plots in Figures 3a-c and 4. The basaltic datasets have an age range of 3.8 to 0 Ga, sourced from the Georoc and EarthChem community data repositories.

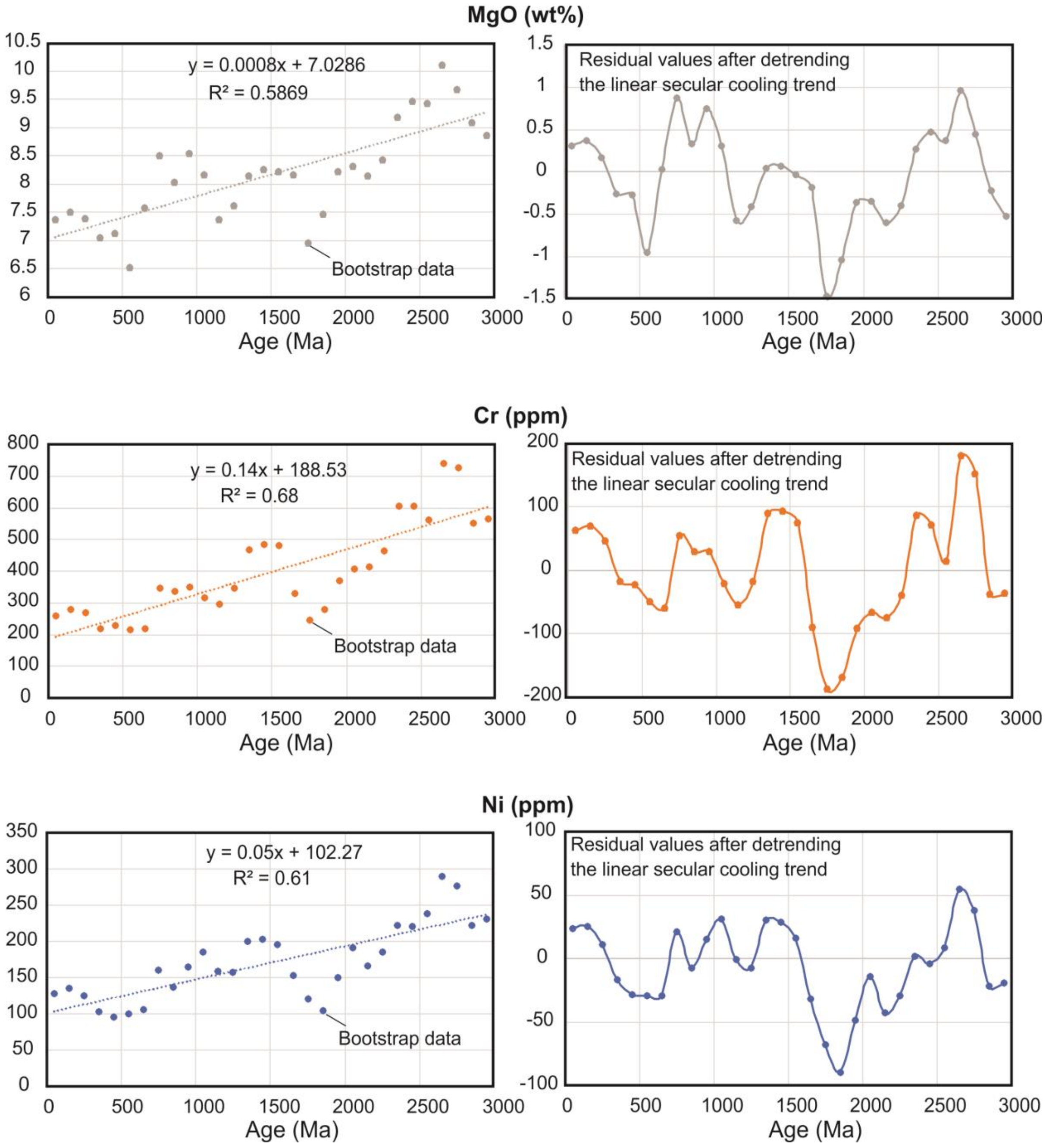


**Supplementary Figure 4**: Variability of global mean of bootstrap data of MgO, Cr and Ni in basalts from the global database (left hand plots), and plots of the same dataset after detrending the linear secular decreases (dashed lines) (right hand plots).

**Supplementary Table 1:** The average bootstrapped values used to create Figures 3, 4 and the normalized values to the secular decrease.

| Age | Ni (ppm) | Cr (ppm) | MgO (wt.%) | Ni secular | Ni subtract | Cr secular | Cr subtract | MgO secular | MgO subtract |
|---|---|---|---|---|---|---|---|---|---|
| **50** | 128.868 | 259.382 | 7.37601 | 104.77 | 24.098 | 195.83 | 63.552 | 7.07 | 0.30601 |
| **150** | 135.395 | 280.1 | 7.51192 | 109.77 | 25.625 | 209.83 | 70.27 | 7.15 | 0.36192 |
| **250** | 125.809 | 269.702 | 7.39924 | 114.77 | 11.039 | 223.83 | 45.872 | 7.23 | 0.16924 |
| **350** | 102.895 | 220.686 | 7.05096 | 119.77 | -16.875 | 237.83 | -17.144 | 7.31 | -0.25904 |
| **450** | 96.6094 | 228.531 | 7.11686 | 124.77 | -28.1606 | 251.83 | -23.299 | 7.39 | -0.27314 |
| **550** | 100.338 | 216.341 | 6.52004 | 129.77 | -29.432 | 265.83 | -49.489 | 7.47 | -0.94996 |
| **650** | 105.476 | 220.801 | 7.58095 | 134.77 | -29.294 | 279.83 | -59.029 | 7.55 | 0.03095 |
| **750** | 161.004 | 347.986 | 8.49647 | 139.77 | 21.234 | 293.83 | 54.156 | 7.63 | 0.86647 |
| **850** | 137.524 | 336.766 | 8.03617 | 144.77 | -7.246 | 307.83 | 28.936 | 7.71 | 0.32617 |
| **950** | 164.648 | 351.43 | 8.53782 | 149.77 | 14.878 | 321.83 | 29.6 | 7.79 | 0.74782 |
| **1050** | 186.009 | 314.992 | 8.17058 | 154.77 | 31.239 | 335.83 | -20.838 | 7.87 | 0.30058 |
| **1150** | 158.908 | 295.723 | 7.37644 | 159.77 | -0.862 | 349.83 | -54.107 | 7.95 | -0.57356 |
| **1250** | 157.666 | 346.648 | 7.61674 | 164.77 | -7.104 | 363.83 | -17.182 | 8.03 | -0.41326 |
| **1350** | 199.933 | 467.83 | 8.1537 | 169.77 | 30.163 | 377.83 | 90 | 8.11 | 0.0437 |
| **1450** | 203.584 | 485.196 | 8.25727 | 174.77 | 28.814 | 391.83 | 93.366 | 8.19 | 0.06727 |
| **1550** | 196.282 | 480.751 | 8.22847 | 179.77 | 16.512 | 405.83 | 74.921 | 8.27 | -0.04153 |
| **1650** | 152.822 | 330.395 | 8.16305 | 184.77 | -31.948 | 419.83 | -89.435 | 8.35 | -0.18695 |
| **1750** | 121.504 | 245.873 | 6.96043 | 189.77 | -68.266 | 433.83 | -187.957 | 8.43 | -1.46957 |
| **1850** | 105.403 | 279.864 | 7.46371 | 194.77 | -89.367 | 447.83 | -167.966 | 8.51 | -1.04629 |
| **1950** | 150.906 | 370.832 | 8.22228 | 199.77 | -48.864 | 461.83 | -90.998 | 8.59 | -0.36772 |
| **2050** | 191.028 | 408.983 | 8.31674 | 204.77 | -13.742 | 475.83 | -66.847 | 8.67 | -0.35326 |
| **2150** | 166.766 | 414.954 | 8.14365 | 209.77 | -43.004 | 489.83 | -74.876 | 8.75 | -0.60635 |
| **2250** | 185.205 | 464.542 | 8.4351 | 214.77 | -29.565 | 503.83 | -39.288 | 8.83 | -0.3949 |
| **2350** | 221.733 | 603.983 | 9.17702 | 219.77 | 1.963 | 517.83 | 86.153 | 8.91 | 0.26702 |
| **2450** | 220.962 | 604.084 | 9.46374 | 224.77 | -3.808 | 531.83 | 72.254 | 8.99 | 0.47374 |
| **2550** | 237.968 | 560.8 | 9.43902 | 229.77 | 8.198 | 545.83 | 14.97 | 9.07 | 0.36902 |
| **2650** | 289.505 | 740.433 | 10.1045 | 234.77 | 54.735 | 559.83 | 180.603 | 9.15 | 0.9545 |
| **2750** | 277.392 | 725.925 | 9.67061 | 239.77 | 37.622 | 573.83 | 152.095 | 9.23 | 0.44061 |
| **2850** | 222.943 | 550.612 | 9.08734 | 244.77 | -21.827 | 587.83 | -37.218 | 9.31 | -0.22266 |
| **2950** | 230.905 | 565.4 | 8.86807 | 249.77 | -18.865 | 601.83 | -36.43 | 9.39 | -0.52193 |
| **3050** | 288.32 | 775.972 | 9.78203 | | | | | | |
| **3150** | 392.479 | 1055.14 | 11.6683 | | | | | | |
| **3250** | 590.063 | 1390.2 | 14.2681 | | | | | | |
| **3350** | 417.756 | 958.609 | 12.1733 | | | | | | |
| **3450** | 367.437 | 921.68 | 10.9689 | | | | | | |
| **3550** | 375.708 | 835.83 | 9.78972 | | | | | | |
| **3650** | 346.972 | 949.448 | 11.7559 | | | | | | |
| **3750** | 305.528 | 945.795 | 11.4923 | | | | | | |
| **3850** | 314.816 | 922.046 | 11.7328 | | | | | | |
| Average >3Ga | 378 | 973 | 12 | | | | | | |
| Average <3Ga | 171 | 400 | 8 | | | | | | |
