# Supplementary material for: Global geochemical fingerprinting of plume intensity suggests coupling with the supercontinent cycle
Source: Nat Commun. 2019 Nov 21;10:5270. doi: 10.1038/s41467-019-13300-4 (PMC6872659; doi:10.1038/s41467-019-13300-4)
Supplement: Supplementary file 3 — Description of Additional Supplementary Files [file 41467_2019_13300_MOESM3_ESM.pdf]

Description of Additional Supplementary Files

File Name: Supplementary Data 1
Description: Datasets of komatiites, picrites, and basaltic rocks used to create Figure 1 and 2.

File Name: Supplementary Data 2
Description: Datasets of basaltic rocks used to create Figure 3 and 4.
