# Supplementary material for: Global geochemical fingerprinting of plume intensity suggests coupling with the supercontinent cycle
Source: Nat Commun. 2019 Nov 21;10:5270. doi: 10.1038/s41467-019-13300-4 (PMC6872659; doi:10.1038/s41467-019-13300-4)
Supplement: Supplementary file 1 — Peer Review File [file 41467_2019_13300_MOESM1_ESM.pdf]

Editorial Note: This manuscript has been previously reviewed at another journal that is not operating a transparent peer review scheme. The manuscript was considered suitable for publication without further review at *Nature Communications*.
